# How does urban blue space affect human well-being? A study based on the stimulus-organism-response theory

**DOI:** 10.3389/fpsyg.2025.1553296

**Published:** 2025-04-09

**Authors:** Pei Lu, Norazmawati Md. Sani, Yuan Li, Yuan Wang

**Affiliations:** ^1^School of Housing, Building and Planning, Universiti Sains Malaysia, Penang, Malaysia; ^2^Department of Life Sciences, Hengshui University, Hengshui, China

**Keywords:** urban blue space, stimulus-organism-response theory, sensory perception, well-being, structural equation modeling

## Abstract

With rapid urbanization and social change, mental health issues have surged. Urban blue spaces (UBSs) offer a potential tool to increase well-being, yet the way in which sensory stimuli shape landscape perception and well-being remains underexplored. Intergenerational integration, a crucial aspect of well-being, refers to shared experiences and social interactions among different age groups, improving cognition and reducing loneliness. However, the role of UBSs in facilitating such interactions remains insufficiently studied. This research, grounded in the Stimulus-Organism-Response (S-O-R) framework, examines how perceived multisensory stimuli (visual, auditory, olfactory, and tactile) influence landscape perception and well-being. Structural equation modeling (SEM) of survey data (*n* = 532) reveals that perceived visual, auditory, and tactile stimuli significantly enhance landscape perception and well-being, while olfactory stimuli have no significant effect. Landscape perception mediates the relationship between visual, auditory, and tactile stimuli and well-being, but not for olfactory stimuli. These findings underscore the importance of optimizing sensory environments in UBSs to enhance psychological restoration. The study provides empirical insights for urban planners and policymakers, advocating for nature-based strategies that enhance visual aesthetics, maintain site quality, integrate natural soundscapes, and improve water accessibility to maximize restorative benefits and foster intergenerational inclusion.

## 1 Introduction

In recent years, mental health issues have surged with rapid urbanization and social change. The World Health Organization (World Health Organization, 2022) reported that nearly one billion people worldwide suffered from anxiety and depression in 2019, with urbanization contributing to their rising prevalence (Lecic-Tosevski, 2019; Ventriglio et al., 2021). In China, rapid urban expansion has reduced access to green and blue spaces, heightening mental health risks (Chen et al., 2020; Gong et al., 2012). Consequently, depression and anxiety now affect 26.4% of the population (Fu et al., 2023), posing a direct threat to well-being (Gautam et al., 2024). In response, researchers increasingly explore Nature-Based Solutions (NBS) to mitigate urbanization’s impact on mental health (Jiang et al., 2020; Li and Lange, 2023; Twohig-Bennett and Jones, 2018).

Since 2000, the European Commission has actively promoted Nature-Based Solutions (NBS), introducing initiatives like “Green Gyms” (Yerrell, 2008) and “Blue Gym” programs (Depledge and Bird, 2009) to enhance mental health through nature. Early research primarily focused on urban green spaces (UGSs)—vegetated areas such as parks, forests, and gardens (Nutsford et al., 2016)—highlighting benefits like reduced disease risk (Villeneuve et al., 2012) and improved mental health (Nguyen et al., 2021). In contrast, studies on urban blue spaces (UBSs) have emerged more recently. Völker and Kistemann (2011) define UBSs as all visible surface waters in cities, including rivers, lakes, and coastal areas. The EU-funded BlueHealth project further describes them as natural or artificial water-dominated environments accessible through direct (e.g., being in, on, or near water) or indirect (e.g., visual or auditory) interaction (Grellier et al., 2017). Notably, UGSs and UBSs often coexist (Smith et al., 2021), and water bodies were historically classified as part of green spaces (Han, 2003; Laumann et al., 2001), leading to an underappreciation of their unique benefits. Recent studies increasingly emphasize UBSs’ impact on psychological health and well-being (Britton et al., 2020; Foley, 2015; White et al., 2020). Like green spaces, UBSs reduce stress, enhance emotional well-being, and support psychological restoration (Gascon et al., 2017). Their natural elements, open views, and tranquil atmosphere further contribute to their restorative potential (White et al., 2020). This study defines UBSs as inland water-centered environments—including rivers, lakes, canals, and wetlands—focusing on their interaction with greenery and human engagement (Foley and Kistemann, 2015; Li and Trivic, 2024).

The World Health Organization defines health as complete physical, mental, and social well-being rather than merely the absence of disease (World Health Organization, 1948). Long-term exposure to UBSs has been linked to improved mental health (Gascon et al., 2017) and reduced psychological distress, particularly in economically disadvantaged communities (Mitchell and Popham, 2008). Beyond individual mental health, UBSs play a crucial role in social cohesion. Compared to green spaces, they are more effective in fostering social interactions and enhancing community well-being (Ashbullby et al., 2013; Foley, 2015; Vaeztavakoli et al., 2018; Völker and Kistemann, 2015). Urban rivers and lakes are natural landscapes and public spaces that encourage intergenerational engagement (Völker and Kistemann, 2011). Hipp et al. (2014) found that UBSs strengthen social connections, while other studies have linked them to a sense of community, neighborhood attachment, and social participation (Liu et al., 2020; Rugel et al., 2017). De Bell et al. (2017) further emphasized their dual role in promoting psychological benefits and social interaction. UBSs also provide targeted benefits for specific groups, including children, adolescents, and older adults (Tillmann et al., 2018; Yeo et al., 2020). However, maximizing their accessibility and usability remains a key challenge (Völker and Kistemann, 2011).

The UN-Habitat (2016) underscores the role of UBSs in fostering sustainable and healthy cities. With over half of the global population living within three kilometers of freshwater bodies (Kummu et al., 2011), their importance in urban planning is increasingly recognized. Effective UBS planning can enhance well-being while advancing urban sustainability. Despite growing evidence of their mental health benefits, limited research has examined how landscape attributes shape perceptions of UBSs and their influence on well-being (Georgiou et al., 2021). Moreover, their role in social integration and community cohesion remains underexplored (Honey-Rosés and Zapata, 2023). While studies on UGSs have assessed youth mental health through intergenerational factors—such as shared use, interaction opportunities, and age-inclusive design (Barron and Rugel, 2023)—the contribution of UBSs to intergenerational well-being is less understood. Additionally, most empirical studies focus on the correlation between waterfront environments and mental health, overlooking the role of sensory stimuli and the mediating effects of individual environmental preferences (Yen et al., 2021). These mediators are critical in enhancing blue health mechanisms yet remain underexamined. To address these gaps, this study focuses on landscape characteristics to evaluate how UBSs influence mental well-being.

UBSs are vital to human health and well-being (Yakinlar and Akpinar, 2022). While rivers and lakes offer restorative benefits, they also pose urban planning challenges. This study examines how landscape perception and perceived multisensory experiences shape well-being and influence user preferences in UBSs. The findings will guide planners and policymakers in designing inclusive, accessible, and engaging blue spaces that maximize public health benefits and urban livability.

## 2 Literature review

### 2.1 The S-O-R theory

Mehrabian and Russell (1974) introduced the Stimulus-Organism-Response (S-O-R) theory, a foundational framework in environmental psychology and behavioral research. This model explains how external stimuli shape behavior through internal psychological states (Buxbaum, 2016). It posits that an individual’s behavior is mediated by emotional and cognitive responses to environmental stimuli: an external stimulus (S) triggers an internal state (O), which subsequently leads to a behavioral response (R) (Mehrabian and Russell, 1974). The S-O-R model evolved from Stimulus-Response (S-R) theory, a behaviorist approach that conceptualized behavior as a direct reaction to external stimuli. However, the S-R theory failed to account for cognitive and emotional processes influencing behavior. To address this, Woodworth introduced the “organism” (O) as a mediating factor in 1929, incorporating cognitive and affective dimensions such as perception, emotion, and motivation (Koppes, 2014). Mehrabian and Russell (1974) refined this framework by integrating a three-dimensional emotional model—dominance, arousal, and delight—expanding its applicability. Later, advancements in cognitive psychology further enhanced the model by incorporating attention, perception, memory, and decision-making (Simon, 1979).

Today, the S-O-R model is widely applied in cognitive psychology, behavioral science, and consumer research. For instance, Tan et al. (2024) used it to analyze how public art attributes influence perceived value and walking behavior. Wu et al. (2021) examined its role in tourism applications, assessing how atmospheric cues shape visitors’ emotional and behavioral responses. Similarly, Chakraborty et al. (2024) explored how tourists’ willingness to pay mediates the relationship between stimuli and sustainable behaviors.

In the context of urban landscape perception, the organism (O) represents cognitive and affective responses to environmental stimuli (S), including visual, auditory, olfactory, and tactile perceptions of UBSs (Völker and Kistemann, 2011). Landscape perception is shaped by sensory inputs, subjective experiences, and cognitive appraisals (Wu et al., 2023; Xie et al., 2024).

Thus, this study applies the S-O-R framework to examine the role of UBSs in shaping well-being. UBSs function as the external stimuli (S), triggering landscape perception (O), which in turn influences self-reported well-being (R). This approach provides insight into how UBSs contribute to psychological health by shaping cognitive and affective responses.

### 2.2 Theoretical framework and current hypotheses

#### 2.2.1 Spatial characteristics (stimulus, S)

As early as 1980, the World Health Organization recognized that perceived environmental quality influences the psychological benefits of UBSs (World Health Organization, 1980). Later, Thompson Coon et al. (2011) underscored the need for further research on its role in well-being. Studies suggest that sensory perceptions beyond visual cues—such as auditory, olfactory, and tactile stimuli—significantly shape well-being and restorative experiences (Holloway and Hubbard, 2014).

Multisensory perception is crucial in optimizing public space experiences by providing appropriate levels of engagement and interest (Haider, 2007; Kaplan et al., 2007; Larkin et al., 2010). The restorative effects of green spaces have been widely studied through a multisensory lens. For example, Zhang et al. (2019) proposed a framework demonstrating how visual, auditory, and tactile perceptions influence restoration via emotional and behavioral regulation. A UK-based qualitative study further explored how multisensory inputs—including visual, auditory, olfactory, and tactile stimuli—shape well-being and social interactions across age groups (Reece et al., 2024).

In this study, spatial characteristics refer to perceived external stimuli that shape human experiences in UBSs, including visual, auditory, olfactory, and tactile elements. This classification is based on Zhang et al. (2019) multisensory perception theory, which extends environmental perception beyond visual cues to incorporate auditory, tactile, and other sensory inputs in shaping landscape experiences and supporting psychological restoration.

While Zhang et al. (2019) primarily examined visual, auditory, and tactile stimuli, emerging research highlights the role of olfactory perception in emotion regulation, memory, and place attachment (Bratman et al., 2024). Investigating olfactory interactions with other sensory inputs may offer a more comprehensive understanding of UBSs’ restorative potential. To enhance this multisensory perspective, this study incorporates olfactory stimuli.

In summary, the current study aims to examine the role of these multisensory landscape elements (e.g., visual, auditory, olfactory, and tactile) as stimuli in enhancing environmental experiences in UBSs and promoting mental health and well-being. Unlike objective measurements (e.g., decibel levels for sound), the analysis relies on subjective sensory perceptions. To ensure clarity, the following section will detail the role of each perceived sensory stimulus in shaping landscape perception and psychological outcomes.

##### 2.2.1.1 Perceived visual stimuli (PVS)

The perceived visual stimuli of UBS play a key role in shaping landscape perception. According to Kaplan and Kaplan’s (1989) landscape preference theory, individuals’ perception and preference for natural environments are shaped by mystery, coherence, legibility, and complexity, which UBSs inherently reflect. Within the visual stimuli perception, openness (Steinwender et al., 2008; Völker and Kistemann, 2011), maintenance (Pflüger et al., 2010), and scenic beauty (Lee L.-H., 2017; Lee L., 2017) are key elements defining UBS landscapes, influencing visual quality, environmental perception, and psychological restoration.

From a landscape perception perspective, expansive water bodies enhance mystery and coherence, encouraging exploration (Herzog, 1985; Kaplan and Kaplan, 1989). Riparian vegetation increases legibility and ecological awareness (Steinwender et al., 2008; Völker and Kistemann, 2011), while well-maintained greenery enhances aesthetic appeal and biophilic engagement (Pflüger et al., 2010, p. 76). Harmonious colors, diverse water features (Jahani and Saffariha, 2020), and biodiversity (e.g., plants, birds, butterflies) (Carrus et al., 2013; Hipp et al., 2016; Hipp and Ogunseitan, 2011) contribute to landscape attractiveness and social connectivity (Hipp et al., 2014; Völker and Kistemann, 2011).

Additionally, visual stimuli support psychological restoration and well-being (Kaplan and Kaplan, 1989; Ulrich et al., 1991). Openness fosters a sense of freedom and security, reducing stress (Völker and Kistemann, 2011). Spacious landscapes promote social interaction and cohesion (Barron and Rugel, 2023), while clean water and orderly landscapes improve accessibility and psychological recovery (Gidlow et al., 2012). Well-maintained environments further reduce visual clutter and anxiety (Pflüger et al., 2010). Thus, we propose the following hypotheses:

***Hypothesis 1 (H1)*** *The perceived visual stimuli of UBSs significantly positively impact landscape perception.*

***Hypothesis 5 (H5)*** *The perceived visual stimuli of UBSs have a significant positive impact on well-being.*

##### 2.2.1.2 Perceived auditory stimuli (PAS)

As an integral aspect of urban environments, soundscapes shape environmental perception (Aletta et al., 2016) and influence spatial mystery and legibility (Pijanowski et al., 2011). Dynamic variations in water sound enhance spatial intrigue and attractiveness (Nicolosi et al., 2021), while people highly value the diversity of water acoustics, from gentle streams to rushing waterfalls, due to their impact on spatial perception (White et al., 2010, p. 490). Additionally, natural sounds, such as flowing water, birdsong, and wind, provide auditory pleasure and enrich environmental complexity, fostering deeper emotional connections to space (White et al., 2010, p. 490; Zhang et al., 2024).

A high-quality soundscape not only enhances environmental perception but also improves well-being. Studies show that natural soundscapes promote mental health, reduce stress, and alleviate anxiety and fatigue (Alvarsson et al., 2010; Guo et al., 2022). Furthermore, the type and compatibility of water sounds affect their restorative potential. Liu et al. (2022) found that river, fountain, and stream sounds provide the highest restorative benefits in UBSs, whereas ocean waves are less effective. In highly urbanized UBSs, integrating natural sounds (e.g., birdsong, flowing water) enhances restoration (Liu et al., 2022) and mitigates the adverse effects of traffic noise (Jeon et al., 2010; You et al., 2010). Additionally, pleasant soundscapes foster psychological well-being, a sense of belonging in public spaces, and social interaction (Abdelmoula et al., 2024), underscoring the positive role of auditory perception in mental health. Based on these findings, we propose the following hypotheses:

***Hypothesis 2 (H2)*** *The perceived auditory stimuli of UBSs significantly positively impact landscape perception.*

***Hypothesis 6 (H6)*** *The perceived auditory stimuli of UBSs have a significant positive impact on well-being.*

##### 2.2.1.3 Perceived olfactory stimuli (POS)

Although often regarded as the least significant sense, olfaction plays a vital role in shaping place attachment (Pennycook and Otsuji, 2015) and influencing spatial perception and environmental quality (Hoover, 2009).

First, olfactory cues can guide movement within a space, shaping landscape preferences by making specific paths or areas more appealing (Hoover, 2009). A diverse range of scents enhances spatial legibility and complexity, enriching environmental experiences (Li et al., 2023). Natural aromas, such as mint, pine, and floral scents, strengthen landscape attachment (Houghton and Houghton, 2013). Moreover, pleasant and enduring scents foster human-environment interactions, encouraging social engagement (Thrift, 2003).

Beyond spatial perception, olfactory stimuli contribute to psychological well-being. Pleasant scents promote relaxation, reduce anxiety, and enhance happiness (Joshi and Hummel, 2024). Natural aromas, such as mint, pine, and floral scents, have been proven to relieve stress and boost well-being (Thrift, 2003). Additionally, they strengthen human-environment connections, support psychological restoration, and alleviate depression (Bratman et al., 2024; Gorman, 2017; Maund et al., 2019). Based on these findings, the following hypotheses are proposed:

***Hypothesis 3 (H3)*** *The perceived olfactory stimuli of UBSs significantly positively impact landscape perception.*

***Hypothesis 7 (H7)*** *The perceived olfactory stimuli of UBSs have a significant positive impact on well-being.*

##### 2.2.1.4 Perceived tactile stimuli (PTS)

Perceived tactile stimuli significantly shape landscape perception by enhancing sensory engagement, promoting physiological stability, and reducing stress, thereby contributing to mental well-being (Warren et al., 2011). UBSs offer direct interaction with water features, such as shallow streams and interactive platforms, fostering place attachment (Asakawa et al., 2004), which can strengthen environmental attachment (Ulrich, 1983; Zhang et al., 2021).

Safety also significantly impacts landscape perception. Fall prevention barriers, adequate lighting, and clear signage enhance spatial legibility and accessibility, encouraging active exploration (Luo et al., 2023; Sonti et al., 2020). Additionally, well-designed pathways, ramps, seating, and shelters promote social inclusion, ensuring diverse user groups can experience UBSs’ restorative benefits (Haeffner et al., 2017; Zhang et al., 2021). Highly accessible blue spaces foster environmental attachment and social interactions, reinforcing their role in urban well-being (Gascon et al., 2017).

Beyond shaping landscape perception, tactile stimuli contribute to mental health by strengthening human-nature connections, promoting relaxation, and reducing anxiety (Ulrich, 1983; Zhang et al., 2021). Safety perception is equally critical; studies suggest that insecure environments diminish restorative benefits, whereas protective infrastructure, such as nighttime lighting, enhances psychological comfort and happiness (Luo et al., 2023; Sonti et al., 2020). Furthermore, high accessibility lowers stress levels and enhances restorative experiences, making blue space relaxation more inclusive (Gascon et al., 2017; Zhang et al., 2021). For older adults and individuals with mobility limitations, inclusive infrastructure ensures equitable access to blue spaces, supporting social well-being (Völker and Kistemann, 2015). Based on the above discussion, the following hypotheses are proposed:

***Hypothesis 4 (H4)*** *The perceived tactile stimuli of UBSs significantly positively impact landscape perception.*

***Hypothesis 8 (H8)*** *The perceived tactile stimuli of UBSs have a significant positive impact on well-being.*

#### 2.2.2 Landscape perception (organism, O)

Individuals develop landscape perception through interaction with their surroundings, shaping subjective preferences based on sensory experiences (Wu et al., 2023). Perception is mediated by the senses, influencing emotional and cognitive responses to UBSs (Völker and Kistemann, 2011). The visual, auditory, olfactory, and tactile characteristics of UBSs contribute to environmental preferences, which are widely studied in environmental psychology, urban planning, and design (Van der Jagt et al., 2014). Various methods have been employed to quantify landscape preferences. For instance, Kaplan’s landscape preference matrix assesses preferences based on mystery, coherence, legibility, and complexity (Kaplan, 1987), which has been applied to measure urban greening preferences in Singapore (Zhao and Zhang, 2024). In contrast, Ning et al. (2024) used a qualitative approach to examine how landscape ecological indicators influence environmental preferences in rural settings.

To systematically assess environmental preferences, Van der Jagt et al. (2014) developed the Environmental Preference Scale (EPS), encompassing four core dimensions: coherence, legibility, complexity, and mystery. Yan et al. (2023) extended this framework to blue spaces by introducing the Water Environment Preference Scale (WEPS), incorporating biophilia and attachment (Kellert and Wilson, 1995; Lewicka, 2011). Given the role of place meaning, identity, and attachment in shaping well-being (Abraham et al., 2010; Davenport and Anderson, 2005; Frumkin, 2003), this study enhances WEPS by integrating place belonging, providing a more comprehensive evaluation of UBS-related psychological and social impacts (Völker and Kistemann, 2011).

Additionally, shared use fosters social integration in public spaces (Nelischer and Loukaitou-Sideris, 2023; Reece et al., 2024). To account for this, we expand WEPS by incorporating a shared-use dimension, resulting in the Blue Environmental Preference Scale (BEPS). This refined scale captures age-group-specific perceptions and preferences for UBSs, offering more profound insights into intergenerational engagement with blue spaces.

According to the Arabian-Russell Model theory, subjective environmental perception plays a pivotal role in health and well-being, influencing emotional states and behavioral choices (Liu et al., 2019; Wang et al., 2019). Perception is a critical mediator between objective environmental quality and health outcomes, as demonstrated by studies on green space exposure and mental well-being (Sallis et al., 2006). Positive perceptions of green spaces have been shown to enhance psychological health (Liu et al., 2019), suggesting a similar mediating effect in UBSs. Based on these findings, the following hypotheses are proposed:

***Hypothesis 9 (H9)*** *Landscape perception has a significant positive impact on well-being.*

***Hypothesis 10 (H10)*** *Landscape perception mediates the effect of perceived visual stimuli in UBS on human well-being.*

***Hypothesis 11 (H11)*** *Landscape perception mediates the effect of perceived auditory stimuli of UBSs on human well-being.*

***Hypothesis 12 (H12)*** *Landscape perception mediates the effect of perceived olfactory stimuli of UBSs on human well-being.*

***Hypothesis 13 (H13)*** *Landscape perceptions mediate the effect of perceived tactile stimuli on human well-being.*

#### 2.2.3 Well-being (reactions, R)

Well-being is a multidimensional construct encompassing mental and psychosocial health, commonly assessed through indicators such as self-esteem, self-efficacy, social confidence, and resilience (Britton et al., 2020). Increasing evidence links environmental health to human well-being (Whitmee et al., 2015), with research suggesting that exposure to UBS can promote pro-environmental behaviors (Alcock et al., 2020) and enhance mental health by reducing stress and anxiety, improving attention restoration, and fostering social interaction across generations (Brito et al., 2019; Dzhambov et al., 2018; Foley and Kistemann, 2015; Pitt, 2018). Schebella et al. (2019) categorized well-being into four key dimensions: stress reduction, mood enhancement, improved concentration, and self-esteem. A study in Hong Kong further examined self-reported health and well-being among older adults in relation to blue space exposure (Garrett et al., 2019). Following these frameworks, this study evaluates the impact of UBSs on well-being across three dimensions: Psychological relief, including stress and anxiety reduction; Cognitive and emotional enhancement, such as improved focus and mood stimulation; Social well-being, including cognitive benefits, reduced loneliness, and intergenerational social engagement. By systematically assessing these dimensions, this study aims to reveal the mechanisms through which UBSs contribute to mental health and social well-being, offering insights for urban planning and public health policies.

### 2.3 The impact of intergenerational factors and related research

Intergenerational integration plays a vital role in enhancing well-being in UBSs. By optimizing spatial diversity, shared accessibility, and multifunctional facilities, UBSs foster interaction among age groups, strengthening social bonds and community cohesion (Barron and Rugel, 2023; Haider, 2007; Mishra et al., 2023; Thang and Kaplan, 2013). Visual, auditory, olfactory, and tactile sensory stimuli are key to promoting intergenerational engagement. Open, multi-layered landscapes attract users of all ages, facilitating cross-generational exchange (Larkin et al., 2010). Dynamic water features, birdsong, and wind sounds create a universally appealing sensory environment (Haider, 2007; Rigolon et al., 2015), while familiar natural scents, such as floral and wetland aromas, enhance emotional connections between generations (Bratman et al., 2024). Additionally, water-friendly designs—including shallow water areas, accessible walkways, and interactive installations—promote safe, inclusive engagement for all age groups (Azevedo, 2020).

Beyond enriching environmental experiences, intergenerational integration contributes to mental health and social well-being, positioning UBSs as vital spaces for fostering cross-generational interactions (Bell et al., 2021). Studies suggest that UBSs enhance social cohesion and community well-being (Barron and Rugel, 2023; Gascon et al., 2017).

While research has explored intergenerational dynamics in urban green spaces and their impact on youth mental health (Barron and Rugel, 2023), studies on UBSs remain limited. Future research should further investigate UBSs’ role in social capital formation, intergenerational communication, and social cohesion (Honey-Rosés and Zapata, 2023). This study integrates key intergenerational factors (e.g., shared use, interaction opportunities, and age-inclusive design) into assessing landscape perception and environmental experience, providing new insights into the relationship between UBSs and human well-being.

### 2.4 Research hypotheses and model building

This study applies the Stimulus-Organism-Response (S-O-R) theory to examine how UBSs influence perceived well-being through sensory perception (visual, auditory, olfactory, and tactile). A theoretical model is proposed to explain the pathway from the environmental multisensory stimuli (S) → landscape perception (O) → well-being (R). The study addresses three key questions:

(1) How do different sensory stimuli shape landscape perception?
(2) How does landscape perception influence well-being?
(3) Is there a mediating effect?

**FIGURE 1:**
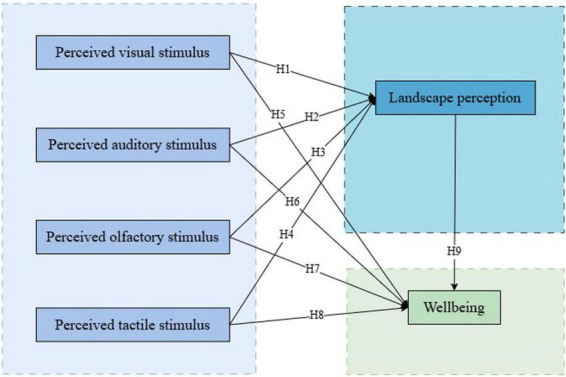
Theoretical model.

Structural Equation Modeling (SEM) is employed to explore these relationships. SEM enables the analysis of multiple variable interactions, assessment of latent constructs, and verification of mediation effects. This approach ensures a rigorous examination of the S-O-R framework, refining theoretical pathways and offering empirical insights to optimize UBS planning for enhanced well-being. Figure 1 illustrates the proposed theoretical model.

## 3 Research design and methodology

### 3.1 Study site

This study focuses on freshwater UBSs, excluding coastal environments such as estuaries. McDougall et al. (2020) noted that urban areas predominantly feature freshwater UBSs, making these findings more generalizable. UBSs are commonly classified into six types: urban rivers, canals, lakes, streams, ponds, and fountains (Völker et al., 2018). Among them, lakes, rivers, and canals are the most significant for enhancing well-being and quality of life (McDougall et al., 2022). Therefore, this study selects three frequently used UBSs—urban rivers, canals, and lakes—as investigation sites.

During March-April 2024, a desk-based review and field surveys were conducted in Hengshui City to identify potential UBSs. The selection criteria required that sites be located within urban environments and frequented by residents. Based on these criteria, the following three UBSs were chosen: People’s Park (urban lake), Fuyang River Ecological Park (urban river), and Ju Wu Canal Ecological Park (urban canal).

**FIGURE 2:**
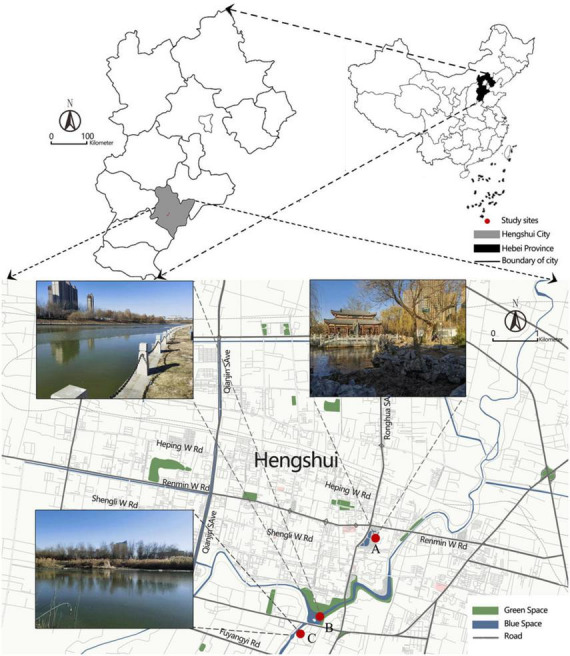
The map and the photos of the study sites: (A) the photo in people’s park (by authors), (B) the photo in Fuyang river (by authors), (C) the photo in Wugong Canal (by authors).

Each UBS site exhibits distinct environmental features, reflecting differences in urbanization, natural elements, and sensory experiences:

• People’s Park (urban lake, Figure 2-A) is surrounded by residential and commercial buildings in the central commercial district. The lake’s varying openness creates a layered spatial experience with diverse visual stimuli. As the city’s oldest park, it holds cultural significance and fosters place attachment. However, due to high foot traffic, its soundscape is dominated by traffic noise and human activity, with minimal natural sounds. It is a highly active UBS, supporting outdoor activities and social interactions.
• Fuyang River Ecological Park (urban river, Figure 2-B) is a historically significant river originating in Fengfeng Mining District, Handan, and flowing into the Bohai Sea. The highly urbanized riverbanks are lined with commercial and residential buildings. The river primarily serves urban drainage functions, limiting green space but offering some openness in visual perception. Its soundscape is shaped by commercial activity and urban noise. The site is used for running and walking, emphasizing functionality and recreation.
• Wugong Canal Ecological Park (urban canal, Figure 2-C) was originally an artificial drainage canal constructed by the Chinese writer Wu Rulun and later restored as a wetland park. It features waterside trails and platforms for fishing, walking, and cycling. The canal has high ecological integrity, with a soundscape dominated by birdsong and flowing water, creating an immersive auditory experience. Additionally, the wetland ecosystem improves air quality, enhancing olfactory perception and reinforcing restorative qualities.

The selection of study sites aligns with the Stimulus-Organism-Response (S-O-R) framework. Each UBS presents distinct variations in sensory stimuli (visual, auditory, olfactory, and tactile), providing an ideal setting for analyzing how sensory experiences shape landscape perception (O) and well-being (R).

### 3.2 Questionnaire design

The first author assembled a panel of five experts from diverse age groups and disciplines, including landscape design, environmental science, ecology, and urban planning, to pilot-test the initial questionnaire on-site. After discussion, items that were ambiguous or difficult to interpret, such as “easy to organize and construct waterfront landscapes,” were removed to enhance clarity and usability. The finalized questionnaire consists of four sections.

The first section collects demographic and socioeconomic data, including gender, age, education, marital status, occupation, and household income. The second section assesses visit frequency and duration at the selected UBSs. The third section evaluates UBSs based on four perceived sensory dimensions—visual, auditory, olfactory, and tactile. Each dimension includes three to four items measuring perceived quality. Visual perception regarding openness, maintenance, and scenic beauty is assessed, reflecting spatial perception and aesthetics. Auditory perception evaluates soundscape quality, loudness, and diversity, capturing acoustic comfort and restorative potential. Based on Li et al. (2023), Olfactory perception considers scent diversity, characteristics, and duration to assess the psychological impact of environmental odors. Tactile perception examines interactivity, safety, and accessibility.

Landscape preferences were measured using BEPS, comprising 15 items categorized into seven factors: coherence, legibility, complexity, mystery, biophilia, attachment, and sharing, with two to three items per factor. Responses were recorded on a five-point Likert scale (1 = strongly disagree, 5 = strongly agree), and scores for each dimension were averaged (Akpinar, 2016).

The final section measures self-reported well-being in UBSs across five dimensions: stress reduction, mood enhancement, concentration, self-esteem (Schebella et al., 2019), and loneliness reduction (Heu and Brennecke, 2023). Participants rated their agreement with statements such as “This place has significantly improved my stress levels” on a five-point Likert scale. To streamline the analysis, stress, and mood were combined into negative mood improvement, attention into positive mood improvement, and self-cognition and loneliness into intergenerational well-being improvement, with scores averaged separately (Akpinar, 2016). Table 1 provides descriptions to aid participant comprehension.

**TABLE 1:** Measurement items.

Constructs	Items	Contents (5-Point Likert Scale)	Preference
Perceived visual stimuli	Openness (PVS1)	The water view here is very open	Yan et al., 2023; Bell et al., 2021; Luo et al., 2023
		This place is very spacious.	
		The proportion of the water body is appropriate here.	
		The sites around the water bodies vary in size.	
	Maintenance (PVS2)	The water here is very clear.	Steinwender et al., 2008; Bell et al., 2021; Lovell et al., 2014; Pflüger et al., 2010
		The facilities here (such as ground, guardrail, etc.) are well maintained.	
		The vegetation here is very well maintained.	
		The facilities here can accommodate people of all ages.	
	Scenic quality (PVS3)	It is a picturesque place.	Lee L.-H., 2017; Lee L., 2017; de Vries et al., 2013, p. 27; Wang et al., 2023; Fisher et al., 2021; Marselle et al., 2015
		The landscape here is very natural, without artificial traces.	
		The color of the vegetation on this site is very harmonious.	
		There are rich species (birds, butterflies, aquatic plants).	
Perceived auditory stimuli	Soundscape (PAS1)	The soundscape here is pleasant.	Pijanowski et al., 2011; Alvarsson et al., 2010; Guo et al., 2022; White et al., 2010, p. 490
	Loudness (PAS2)	Volume is soft.	
	Diversity of sounds (PAS3)	The soundscape here is varying (Such as bird song, wind, water wave beating sound)	
Perceived olfactory stimuli	Diversity of scents (POS1)	The air here is filled with various scents, such as the fragrance of flowers and fresh grass.	Bratman et al., 2024; Maund et al., 2019
	Scent characteristic (POS2)	The scent here is unique.	
	Duration (POS3)	The fragrance here has a long duration.	
Perceived tactile stimuli	Interactivity (PTS1)	It is easy to get to the waterfront.	Zhang et al., 2021; Ulrich, 1983; Bell et al., 2021
		You can feel the wind near the water here.	
		Various water-based or waterside activities, such as boating and fishing, can be enjoyed here.	
	Safety (PTS2)	There is adequate protection against falling water.	Luo et al., 2023
		Lighting is adequate in this area.	
	Accessibility (PTS3)	There is a barrier-free design?	Völker and Kistemann, 2015; Gascon et al., 2017
		It is very convenient to get to here.	
Landscape perception	Coherence (LP1)	The style here is very consistent with the surrounding environment.	Van der Jagt et al., 2014
		The different landscapes here blend perfectly.	
	Legibility (LP2)	It is easy to find my way around this waterfront space.	Van der Jagt et al., 2014
		The position of different landscape elements is apparent.	
	Complexity (LP3)	This scene contains a lot of different elements.	Van der Jagt et al., 2014
		This waterfront landscape looks changeful.	
	Mystery (LP4)	This waterfront view looks secluded and profound.	Van der Jagt et al., 2014
		This waterscape could arouse my interest in further exploration.	
	Biophilia (LP5)	This waterfront landscape has a sense of vitality.	Kellert and Wilson, 1995; Lewicka, 2011
		This waterfront space allows me to get very close to nature.	
	Attachment (LP6)	I want to revisit this waterfront space.	Kellert and Wilson, 1995; Lewicka, 2011
		It gives me a strong sense of local identity.	
	Sharing (LP7)	It allows me to build strong social connections with others.	Nelischer and Loukaitou-Sideris, 2023; Reece et al., 2024
		There are opportunities for people of different ages to communicate and interact, such as familiar games and observation experiences.	
		Here, people of different ages are connected in sight.	
Well-being	Improvement of negative emotion (WB1)	I think a typical visit to this blue space can significantly reduce my stress.	Schebella et al., 2019
		I think a typical visit to this blue space can improve my negative mood.	
	Improvement of positive emotions (WB2)	I think a typical visit to this blue space can significantly improve my attention.	Schebella et al., 2019
	Inter-generational positive effects (WB3)	I think a typical visit to this blue space can significantly improve my cognitive skills.	Barron and Rugel, 2023; Nelischer and Loukaitou-Sideris, 2023
		I think sharing this blue space with other age groups can relieve my loneliness greatly.	

### 3.3 Data collection

The survey was conducted in Hengshui from October 1 to November 20, 2024, targeting adults aged 18 and above. To ensure data quality, six trained student surveyors from Hengshui University, the first author’s institution, were recruited and received a half-day training session before data collection. At each study site, systematic random sampling was employed to ensure a representative selection of participants. Surveyors followed a predetermined interval strategy, inviting every odd-numbered visitor rather than selecting respondents based on convenience. This method minimized selection bias and improved sample representativeness. The questionnaire required approximately 5–8 min to complete. As a token of appreciation, participants received small incentives, such as dishwashing liquid or noodles, upon survey completion.

A total of 625 questionnaires were collected, of which 532 were deemed valid after excluding incomplete or inconsistent responses, yielding an effective response rate of 85%. The sample size exceeds the recommended threshold of ten times the number of questionnaire items (22), meeting the requirements for SEM (Jackson, 2003).

Data were analyzed using SEM, a robust statistical method widely applied in social sciences, management, and psychology (Hair et al., 2011; Sarstedt et al., 2021). SEM enables examining relationships between latent and observed variables, making it particularly effective for assessing complex inter-variable associations. This study includes reliability and validity analysis of the measurement model, followed by structural model testing to evaluate hypothesized relationships. Statistical significance was set at *p* < 0.05, with additional thresholds of *p* < 0.01 and *p* < 0.001 where applicable.

### 3.4 Descriptive statistics

Table 2 presents the sample’s demographic, economic, and behavioral characteristics. Female respondents (62%) outnumber males (38%), and most (62.8%) are over 60. Marital status is nearly balanced (52.1% unmarried, 47.9% married). Education levels vary, with 57.7% holding a bachelor’s or college degree, while only 0.2% have a doctorate. Household income is primarily between RMB 2501-5000 (30.1%), with 26.1% earning below RMB 2500. Regarding UBS visits, 54.1% visit rarely, 18.2% daily, and 23% at least weekly. Most visits last 30–60 min (26.1%) or 15–30 min (24.6%). These findings provide a foundation for analyzing UBS’s perception and well-being.

**TABLE 2:** Descriptive statistics results.

Sample	Category	Number	Percentage
Gender	Male	202	38%
	Female	330	62%
Age	18–44	117	22%
	45–60	81	15.20%
	Over 60	334	62.80%
Marital Status	Unmarried	277	52.10%
	Married	255	47.90%
Education	Junior high school or below	94	17.70%
	High school	89	16.70%
	Bachelor’s degree or college	307	57.70%
	Master’s degree	41	7.70%
	Doctor or above	1	0.20%
Monthly household income (RMB)	Under 2500	139	26.10%
	2501–5000	160	30.10%
	5001–7500	135	25.40%
	7501–10000	56	10.50%
	Above 10000	42	7.90%
Frequency of visits	Seldom	288	54.10%
	Once per month	25	4.70%
	Once per week	60	11.30%
	Several times per week	62	11.70%
	Every day	97	18.20%
Length of visit	<15 min	33	6.20%
	15–30 min	131	24.60%
	30 min–1 h	139	26.10%
	1–2 h	129	24.20%
	>2 h	100	18.80%

## 4 Data analysis and results

### 4.1 Measurement model analysis

#### 4.1.1 Reliability test

Reliability analysis was conducted using SPSS 27.0, with results presented in Table 3. The Cronbach’s α-values ranged from 0.863 to 0.944, all exceeding the 0.7 thresholds (Hair et al., 1986), confirming high internal consistency. Deleting any item did not improve Cronbach’s α, and Corrected Item-to-Total Correlation (CITC) values remained above 0.5, further supporting scale reliability (Churchill, 1979).

**TABLE 3:** Results of confidence analysis.

Dimension	Items	Corrected item-to-total correlation	Cronbach’s α if Item deleted	Cronbach’s α
PVS	PVS1	0.732	0.882	0.886
	PVS2	0.827	0.797	
	PVS3	0.781	0.837	
PAS	PAS1	0.836	0.819	0.897
	PAS2	0.807	0.844	
	PAS3	0.749	0.894	
POS	POS1	0.762	0.840	0.882
	POS2	0.806	0.801	
	POS3	0.745	0.856	
PTS	PTS1	0.706	0.838	0.863
	PTS2	0.746	0.805	
	PTS3	0.774	0.776	
LP	LP1	0.755	0.94	0.944
	LP2	0.797	0.937	
	LP3	0.847	0.932	
	LP4	0.798	0.936	
	LP5	0.841	0.933	
	LP6	0.811	0.935	
	LP7	0.847	0.933	
WB	WB1	0.753	0.855	0.884
	WB2	0.829	0.785	
	WB3	0.745	0.863	

#### 4.1.2 Validity test

Validity was assessed using the Kaiser-Meyer-Olkin (KMO) test and Bartlett’s test of sphericity in SPSS 27.0 (Table 4). KMO values exceeded 0.5 (ranging from 0.723 to 0.934), and Bartlett’s test was statistically significant (*p* < 0.05), indicating suitability for factor analysis (Kaiser, 1974). Principal component analysis extracted a single factor per variable with eigenvalues >1, and cumulative variance contribution exceeded 50%, demonstrating high explanatory power. Factor loadings were >0.6, and communalities exceeded 0.5, aligning with prior research (Kohli et al., 1998).

**TABLE 4:** Analysis results of exploratory factor.

Dimension	Items	KMO	Bartlett Sphere test	Factor loading	Commonality	Eigenvalue	Total variation explained
PVS	PVS1	0.727	<0.001	0.876	0.768	2.451	81.69%
	PVS2			0.929	0.862		
	PVS3			0.906	0.821		
PAS	PAS1	0.735	<0.001	0.931	0.867	2.489	82.97%
	PAS2			0.917	0.841		
	PAS3			0.884	0.782		
POS	POS1	0.736	<0.001	0.896	0.802	2.428	80.93%
	POS2			0.918	0.843		
	POS3			0.885	0.783		
PTS	PTS1	0.730	<0.001	0.866	0.751	2.359	78.63%
	PTS2			0.889	0.791		
	PTS3			0.904	0.817		
LP	LP1	0.934	<0.001	0.818	0.670	5.250	75.00%
	LP2			0.852	0.726		
	LP3			0.891	0.794		
	LP4			0.854	0.730		
	LP5			0.888	0.788		
	LP6			0.865	0.748		
	LP7			0.891	0.795		
WB	WB1	0.723	<0.001	0.890	0.793	2.439	81.29%
	WB2			0.930	0.864		
	WB3			0.884	0.782		

Convergent validity was assessed using AMOS. Following established criteria, standardized factor loadings should exceed 0.5, composite reliability (CR) should be ≥ 0.7 (Hair et al., 2020), and the average variance extracted (AVE) should be >0.5 (Hair et al., 2021). As shown in Table 5, all factor loadings exceed 0.7, CR values surpass 0.8, and AVE values are above 0.6, confirming strong convergent validity.

**TABLE 5:** Analysis results of convergent validity.

Dimension	Items	Unstandardized factor loading	Standardized factor loading	S.E.	*p*-value	AVE	CR
PVS	PVS1	1.000	0.779	–	–	0.729	0.889
	PVS2	1.079	0.896	0.049	0.000		
	PVS3	1.062	0.882	0.048	0.000		
PAS	PAS1	1.000	0.921	–	–	0.748	0.899
	PAS2	0.958	0.872	0.034	0.000		
	PAS3	0.869	0.798	0.036	0.000		
POS	POS1	1.000	0.834	–	–	0.717	0.883
	POS2	1.086	0.898	0.048	0.000		
	POS3	1.01	0.805	0.048	0.000		
PTS	PTS1	1.000	0.783	–	–	0.682	0.865
	PTS2	1.184	0.824	0.061	0.000		
	PTS3	1.137	0.869	0.056	0.000		
LP	LP1	1.000	0.785	–	–	0.700	0.942
	LP2	0.996	0.819	0.047	0.000		
	LP3	1.093	0.869	0.048	0.000		
	LP4	1.048	0.839	0.049	0.000		
	LP5	1.121	0.908	0.049	0.000		
	LP6	1.116	0.828	0.051	0.000		
	LP7	1.036	0.804	0.044	0.000		
WB	WB1	1.000	0.869	–	–	0.746	0.898
	WB2	1.144	0.879	0.047	0.000		
	WB3	1.048	0.843	0.049	0.000		

Discriminant validity was evaluated by comparing AVE square roots with construct correlations (Fornell and Larcker, 1981). As presented in Table 6, AVE square roots exceeded inter-construct correlations, confirming discriminant validity. Additionally, Heterotrait-Monotrait (HTMT) ratios remained <0.90, further supporting discriminant validity (Henseler et al., 2015; Table 7).

**TABLE 6:** Discriminant validity.

	PVS	PAS	POS	PTS	LP	WB
PVS	**0.854**					
PAS	0.478	**0.865**				
POS	0.283	0.205	**0.847**			
PTS	0.486	0.439	0.271	**0.826**		
LP	0.633	0.575	0.293	0.539	**0.837**	
WB	0.465	0.440	0.230	0.421	0.520	**0.948**

The bolded part of the diagonal line indicates the square root of AVE.

**TABLE 7:** Heterotrait–monotrait (HTMT) ratio.

	PAS	LP	POS	PTS	PVS	WB
PAS						
LP	0.626					
POS	0.231	0.321				
PTS	0.500	0.597	0.312			
PVS	0.537	0.692	0.321	0.558		
WB	0.495	0.571	0.26	0.483	0.529	

### 4.2 Structural model analysis

SEM was conducted following Kline (2023) fit indices criteria: χ^2^/df < 5, RMSEA & SRMR < 0.08, NFI, GFI, IFI, TLI, and CFI > 0.90. All model fit indices met these standards (Table 8), confirming strong structural validity. To assess common method bias, a common method factor was introduced (Xiong et al., 2012). Two models were compared: M1 (without a common method factor) and M2 (with a common method factor). Although M2 showed a slightly improved fit, the changes were negligible (RMSEA and SRMR differences <0.05; NFI, GFI, IFI, TLI, and CFI differences <0.1), suggesting common method bias was effectively controlled (Table 8).

**TABLE 8:** Model fit measures, comparison of model fit between M1 and M2.

Common Indices	χ^2^/df	RMSEA	GFI	NFI	IFI	TLI	CFI	SRMR
Judgment criteria	<5	<0.08	>0.9	>0.9	>0.9	>0.9	>0.9	<0.08
CFA value	2.705	0.057	0.917	0.942	0.963	0.956	0.963	0.034
CCLFM value	2.411	0.052	0.926	0.949	0.969	0.963	0.969	0.033

**FIGURE 3:**
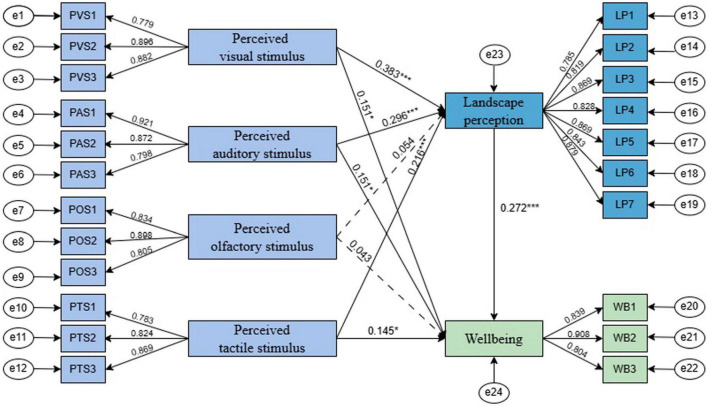
Model path analysis results. *p < 0.05, ***p < 0.001.

Moreover, path analysis results (Figure 3 and Table 9) revealed significant relationships except for H3 and H7, reinforcing the model’s explanatory power regarding UBSs’ well-being.

**TABLE 9:** Hypothesis test results.

Hypothesis	Relationship	Unstd.	Std.	*SE*	*p*-values	Support→
H1	PVS→LP	0.355	0.383	0.044	0.000	Yes
H2	PAS→LP	0.255	0.296	0.037	0.000	Yes
H3	POS→LP	0.043	0.054	0.028	0.127	No
H4	PTS→LP	0.196	0.216	0.04	0.000	Yes
H5	PVS→WB	0.159	0.151	0.065	0.014	Yes
H6	PAS→WB	0.148	0.151	0.053	0.005	Yes
H7	POS→WB	0.039	0.043	0.039	0.321	No
H8	PTS→WB	0.150	0.145	0.057	0.009	Yes
H9	LP→WB	0.310	0.272	0.074	0.000	Yes

### 4.3 Analysis of mediating effects

Mediation effects were tested using 1,000 bootstrap resamples. As shown in Table 10, LP significantly mediated the effects of PVS, PAS, and PTS on WB (*p* < 0.05, confidence intervals excluding zero). However, POS had no significant direct or indirect effect, indicating its negligible role in the model.

**TABLE 10:** Results of mediated path tests.

Effect	Parameter	Estimate	95% confidence interval		
			***p*-value**	**Lower**	**Upper**
Indirect effect	PVS→LP→WB	0.104	0.015	0.015	0.288
	PAS→LP→WB	0.080	0.013	0.015	0.221
	POS→LP→WB	0.015	0.079	−0.002	0.060
	PTS→LP→WB	0.059	0.015	0.011	0.179
Direct effect	PVS→WB	0.151	0.179	−0.077	0.38
	PAS→WB	0.151	0.164	−0.06	0.363
	POS→WB	0.043	0.351	−0.045	0.138
	PTS→WB	0.145	0.102	−0.023	0.381
Total effect	PVS→WB	0.255	0.036	0.025	0.497
	PAS→WB	0.232	0.008	0.057	0.429
	POS→WB	0.057	0.219	−0.035	0.156
	PTS→WB	0.204	0.037	0.026	0.402

## 5 Discussion

Of the 13 tested hypotheses, 10 were supported, while H3, H7, and H12 were not. H1, H2, H4, and H9 demonstrated the strongest effects.

### 5.1 Hypotheses for spatial characteristics and landscape perception of UBSs

The results show that perceived visual, auditory, and tactile stimuli in UBSs significantly influence landscape perception, with standardized path coefficients of 0.383, 0.296, and 0.216, respectively (*p* < 0.001). This indicates that a one-unit increase in these stimuli enhances residents’ environmental perception by 0.383, 0.296, and 0.216 standard deviations, supporting H1, H2, and H4. Among them, visual stimuli have the strongest effect, suggesting that optimizing UBSs’ visual environment is the most effective way to enhance landscape perception (Deng et al., 2020). Conversely, perceived olfactory stimuli do not significantly affect landscape perception (β = 0.054, *p* > 0.05), leading to the rejection of H3. This may be due to the relatively weak olfactory stimuli in UBSs, as olfaction generally requires a more intense trigger to be noticeable. Moreover, vision is the dominant sense in environmental perception, while olfaction is often secondary (Pennycook and Otsuji, 2015). Studies have shown that people rely more on vision when perceiving environments and pay less attention to olfaction (Wang et al., 2023), which explains why perceived olfactory stimuli have a weaker influence on landscape preference perception in UBSs.

### 5.2 Hypotheses for spatial characteristics and well-being of UBSs

Similarly, perceived visual, auditory, and tactile stimuli positively impact well-being, with standardized path coefficients of 0.151, 0.151, and 0.145, respectively (*p* < 0.05), confirming H5, H6, and H8. This suggests that enhancing UBSs’ visual, auditory, and tactile environments can promote relaxation and stress relief, ultimately improving well-being (Whitmee et al., 2015). However, the effect of perceived olfactory stimuli on well-being is not significant (β = 0.043, *p* > 0.05), invalidating H7. A possible explanation is that the study was conducted in a northern Chinese city during autumn and winter when plant diversity, a key contributor to olfactory experiences, is limited (He et al., 2022). Consequently, olfactory perception in UBSs may be weaker during this period, reducing its impact on well-being.

### 5.3 Hypotheses for landscape perception and well-being of UBSs

The results indicate that natural landscape perception significantly enhances well-being (β = 0.272, *p* < 0.001), meaning a one-unit increase in perception intensity leads to a 0.272-unit rise in well-being. This supports H9, suggesting that more profound engagement with UBS environments enhances individuals’ appreciation of their uniqueness, thereby amplifying well-being (Liu et al., 2019; Wang et al., 2019).

**FIGURE 4:**
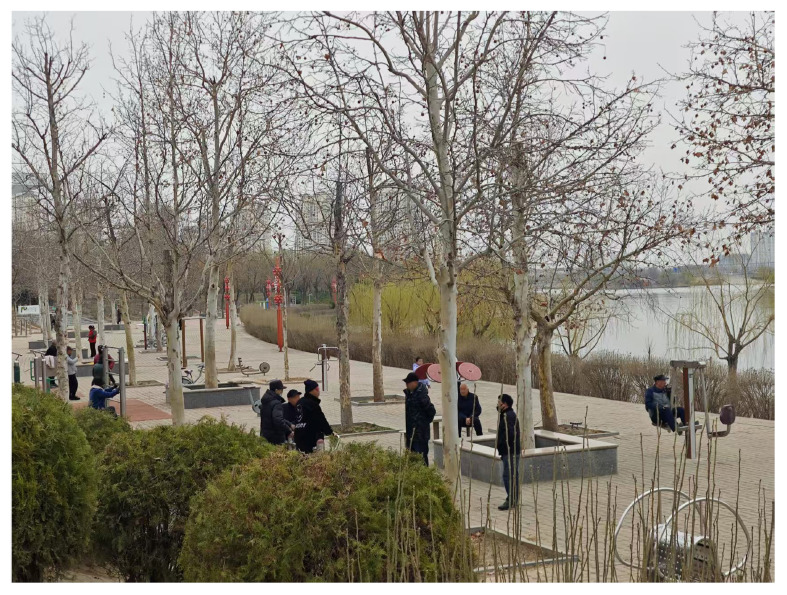
Shared spaces in UBS facilitating social interaction (by authors).

**FIGURE 5:**
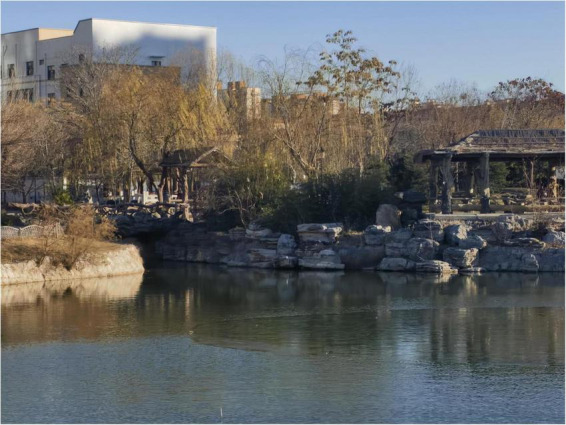
Landscape complexity and biophilic elements in UBS: diverse vegetation and waterbody structures (by authors).

Additionally, all seven dimensions of landscape perception—coherence, legibility, complexity, mystery, biophilia, attachment, and sharing—contributed significantly to overall perception, though with varying degrees of influence (β = 0.785 –0.879). Sharing (β = 0.879), complexity (β = 0.869), and biophilia (β = 0.869) had the strongest effects, highlighting the importance of social interaction, moderate visual complexity in water features, and proximity to nature in shaping UBS perceptions (Kaplan and Kaplan, 1989; Ulrich, 1983). As illustrated in Figure 4, UBSs promote social interaction through shared spaces such as seating areas and open gathering zones, reinforcing the impact of sharing on landscape perception. Figure 5 highlights the contributions of complexity and biophilia, showcasing diverse vegetation, varied waterbody structures, and rich biodiversity, all of which enhance engagement and psychological restoration. Attachment (β = 0.843) and mystery (β = 0.828) were also influential, underscoring the role of emotional connection and exploratory elements in enriching perception. Conversely, coherence (β = 0.785) and legibility (β = 0.819) had relatively lower contributions, potentially due to the dominance of natural aesthetics over structural order and navigability. These findings emphasize the need for UBS designs to prioritize shared experiences, biodiversity, and visually engaging water features while also fostering emotional attachment and functional usability (Van den Berg et al., 2007; White et al., 2013).

Regarding well-being, the impact of intergenerational benefits (β = 0.804) was lower than that of stress reduction, mood improvement, and attention enhancement. This may reflect a tendency for individuals to prioritize immediate psychological relief in UBS settings. In contrast, long-term benefits, such as enhanced self-awareness and reduced loneliness through social interaction, are more likely to manifest over extended social engagement.

### 5.4 Hypotheses for spatial characteristics, landscape perception, and well-being of UBSs

Building on these findings, we further examined whether landscape perception mediates the relationship between sensory stimuli and well-being.

The analysis revealed significant indirect effects of perceived visual stimuli (PVS), auditory stimuli (PAS), and tactile stimuli (PTS) on well-being (WB), with effect values of 0.104, 0.080, and 0.059, respectively. The bias-corrected 95% confidence intervals did not contain zero, and all *p*-values were below 0.05, confirming that landscape perception significantly mediated these relationships. However, the indirect effect of perceived olfactory stimuli on well-being was 0.015, with a confidence interval including zero and a *p*-value exceeding 0.05, indicating no significant mediation effect.

The direct effects of PVS, PAS, POS, and PTS on WB were 0.151, 0.151, 0.043, and 0.145, respectively, but none were statistically significant, as their confidence intervals included zero and *p*-values exceeded 0.05. Similarly, the total effects of PVS, PAS, and PTS on WB were significant (0.255, 0.232, and 0.204, respectively), while POS showed no significant total effect (0.057). These findings confirm hypotheses H10, H11, and H13 but reject H12, reinforcing that landscape perception serves as the primary mediating mechanism between sensory stimuli (excluding olfactory) and well-being.

The results suggest that perceived visual, auditory, and tactile elements in UBSs substantially enhance well-being through landscape perception, with no significant direct effects. In contrast, perceived olfactory stimuli had minimal influence on all pathways. This may be attributed to the lower intensity of plant-derived odors and reduced air humidity in northern China during autumn and winter (Washington et al., 2019). While visual (e.g., water bodies and greenery) and auditory elements (e.g., bird songs and flowing water) effectively stimulate landscape perception and improve well-being (Alvarsson et al., 2010; Deng et al., 2020; Guo et al., 2022), olfactory experiences rely on specific environmental factors such as aromatic vegetation. The seasonal decline in plant emissions weakens olfactory stimulation (He, 2022), diminishing its impact on landscape perception and well-being (Wang et al., 2023).

Given that landscape perception mediates the relationship between sensory stimuli and well-being, with no significant direct effects observed, these findings align with the indirect effect theory in environmental psychology, which posits that sensory experiences influence well-being primarily through psychological states such as preference and cognition (Kaplan, 1995; Liu et al., 2019). Therefore, optimizing UBSs in northern China should prioritize enhancing visual and auditory elements. Strategies include improving water feature aesthetics, refining soundscape design, incorporating aromatic vegetation to strengthen olfactory stimulation, and organizing multisensory activities such as seasonal sightseeing events or concerts (Washington et al., 2019).

### 5.5 Sample representation

While the above findings highlight the effects of sensory stimuli on landscape perception and well-being, it is essential to consider sample representativeness to assess the broader applicability of these results.

Table 2 presents the sample’s demographic characteristics and highlights potential representational biases. Most respondents were female (62%), with 62.8% aged over 60. This aligns with some previous studies indicating that older adults are primary users of UBSs, particularly during weekdays and off-peak hours (Pleson et al., 2014; Yang et al., 2023; Yung et al., 2019). Younger adults (18–44 years) accounted for 22%, while the 45–60 age group represented 15.2%, suggesting a relatively lower representation of younger adults in this study. Regarding UBS visitation frequency, 54.1% of respondents reported infrequent visits, whereas 18.2% visited daily, and 27.7% visited at least once monthly. This distribution reflects real-world UBS usage patterns shaped by accessibility, lifestyle, and leisure preferences.

While the sample provides valuable insights into adult perceptions of UBSs, its composition may limit the generalizability of findings, particularly for younger populations. Future studies should employ stratified sampling and targeted outreach to achieve a more balanced age distribution and better collect data during different periods (e.g., weekends) to capture the experiences of younger and working populations. Additionally, longitudinal studies could assess the stability of UBS-related well-being outcomes across different user groups, enhancing the broader applicability of the findings.

## 6 Conclusion

### 6.1 Contribution

This study advances the S-O-R framework by systematically integrating multisensory perception (visual, auditory, olfactory, and tactile) into analyzing UBSs and their effects on well-being. While prior research has primarily focused on visual perception, this study highlights the interplay of multiple sensory stimuli, providing a more comprehensive understanding of environmental influences on human well-being. Additionally, by incorporating intergenerational integration into well-being assessments, this study underscores UBS’s role in fostering social interaction and cohesion, offering actionable insights for urban planning and sustainable development.

#### 6.1.1 Theoretical contributions

This study fills a critical gap in environmental psychology and urban planning by moving beyond visual-dominant approaches and incorporating multisensory perception into studying UBSs. It further extends the indirect effect theory by empirically confirming that landscape perception mediates the relationship between sensory stimuli and well-being. It demonstrates that well-being is primarily shaped by cognitive and emotional responses rather than direct physiological effects.

These findings contribute to a deeper understanding of how multisensory perception enhances psychological restoration, offering a theoretical foundation for optimizing urban nature-based solutions. Additionally, by integrating intergenerational interaction into well-being assessments, this study provides novel insights into how UBSs contribute to social cohesion, supporting inclusive urban design and policymaking.

#### 6.1.2 Management contributions

This study provides practical recommendations for urban planners, policymakers, social workers, and community administrators by identifying key environmental factors that enhance UBSs’ well-being. The findings emphasize the importance of multisensory optimization in UBS planning and management:

• Visual Environment: In addition to enhancing spatial openness, improving landscape maintenance, aesthetic appeal, and biodiversity strengthens visual engagement and psychological restoration.
• Auditory Environment: Incorporating natural soundscapes (e.g., birdsong, flowing water) and regulating sound levels enhances acoustic comfort and fosters emotional well-being.
• Olfactory Environment: The intensity, persistence, and diversity of scents shape olfactory perception. Strategies such as planting aromatic vegetation, optimizing water circulation, and improving airflow can strengthen sensory appeal.
• Tactile Experience: Enhancing water accessibility (e.g., walkways, open shorelines) and integrating interactive elements (e.g., shallow water areas) encourage user engagement, while safety measures (e.g., barriers, lighting, universal design) ensure inclusivity and accessibility.

From a policy perspective, these findings offer a scientific basis for:

• Optimizing UBS development by integrating sustainable resource allocation and evidence-based maintenance strategies.
• Creating age-inclusive UBS designs that enhance shared experiences and psychological well-being, which may contribute to intergenerational integration.
• Addressing demographic shifts by enhancing universal accessibility, ensuring UBSs remain socially and physically inclusive.

By bridging academic research and practical applications, this study equips decision-makers with evidence-based strategies for designing more inclusive, engaging, and sustainable UBSs that optimize both individual well-being and social cohesion.

### 6.2 research limitations and future research directions

Despite its contributions, this study has several limitations that offer avenues for future research:

Geography: This study is based on data collected in Hengshui, China, which may limit the generalizability of the findings to other regions. Cultural, economic, and policy differences can influence UBS experiences, necessitating broader investigations across diverse geographic contexts. Future research could expand data collection to multiple regions, incorporating cross-cultural comparisons or multi-regional studies to assess the impact of regional variations on UBS perception and well-being.

Study Design: The study employs a cross-sectional design, capturing relationships between variables at a single point in time but lacking temporal insights into evolving perceptions of UBSs. Additionally, seasonal variations in UBS experiences were not explicitly examined. Future studies should consider longitudinal designs or multi-wave cross-sectional surveys to track temporal dynamics, capturing how seasonal changes and prolonged exposure influence well-being outcomes. While systematic random sampling was employed, potential biases may arise due to time-specific visitation patterns and voluntary participation. Future studies could implement stratified sampling across different periods and days of the week to further enhance sample representativeness.

Sample Representativeness and Age Distribution: This study focuses on adults (18+ years), excluding minors whose psychological responses to UBSs may differ due to developmental and behavioral variations. Adolescents, in particular, exhibit distinct environmental perceptions and participation patterns. Future research should incorporate younger age groups to enable cross-age comparisons and explore age-specific preferences and impacts on well-being in UBS settings. However, the sample structure presents certain biases, with over 60% of respondents being older adults, while the 18–44 age group is underrepresented. Furthermore, more than 50% of participants reported infrequent UBS visits, which may affect the interpretation of findings. Given these limitations, the study’s generalizability requires careful consideration. Future research should refine sampling strategies to achieve a more balanced age distribution, employing stratified sampling to ensure proportional representation across age groups. Data collection should also target weekends and holidays to increase young adult participation. Moreover, multi-stage sampling can be used to ensure adequate representation of individuals with varying UBS visitation frequencies, thereby enhancing the study’s robustness and applicability. Research methods: While quantitative methods systematically analyze variable relationships, they fail to capture the nuanced individual factors shaping adults’ UBS preferences. Future research should integrate qualitative approaches to uncover deeper insights, forming a more comprehensive analytical framework.

Addressing these limitations through expanded sample scope, longitudinal approaches, and mixed-methods research will enhance the generalizability and depth of UBS research. Future investigations should adopt interdisciplinary perspectives to optimize UBS design and maximize its psychological and social benefits.

## Data Availability

The raw data supporting the conclusions of this article will be made available by the authors, without undue reservation.
